# Diethyl [(9-anthr­yl)(4-methyl­anilino)meth­yl]phospho­nate

**DOI:** 10.1107/S1600536811025943

**Published:** 2011-07-09

**Authors:** Ivanka Kraicheva, Ivelina Tsacheva, Elitsa Vodenicharova, Emil Tashev, Kolio Troev

**Affiliations:** a Institute of Polymers, Bulgarian Academy of Sciences, Acad. G. Bonchev str., bl. 103A, 1113 Sofia, Bulgaria

## Abstract

The title compound, C_26_H_28_NO_3_P, crystallized with two independent mol­ecules in the asymmetric unit. The structural features (bond lengths and angles) of the two mol­ecules are almost identical. The inter­planar angle between the anthracene and toluidine rings is similar in the two mol­ecules, with values of 82.92 (5) and 80.70 (5)°. In the crystal, both molecules form inversion dimers linked by pairs of N—H⋯O hydrogen bonds. Three of the four ethyl groups are disordered over two sets of sites, the major components having occupancies of 0.748 (15), 0.77 (4) and 0.518 (19).

## Related literature

For general background of the use of amino­phospho­nic acid derivatives in organic synthesis and as biologically active compounds, see: Kraicheva *et al.* (2011 ▶).
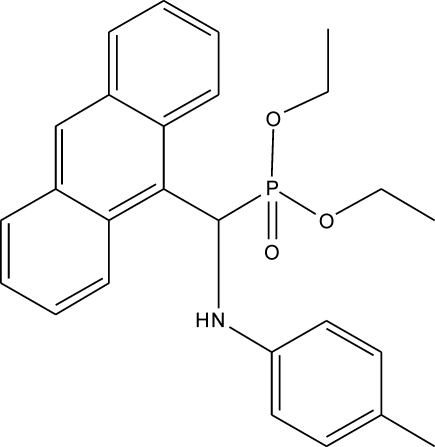


## Experimental

### 

#### Crystal data

C_26_H_28_NO_3_P
*M* *_r_* = 433.46
Triclinic, 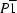

*a* = 9.5990 (3) Å
*b* = 12.6386 (5) Å
*c* = 20.2131 (7) Å
α = 75.865 (3)°
β = 76.780 (4)°
γ = 74.927 (3)°
*V* = 2260.13 (14) Å^3^
*Z* = 4
Mo *K*α radiation
μ = 0.15 mm^−1^
*T* = 290 K
0.31 × 0.18 × 0.15 mm

#### Data collection

Agilent SuperNova Dual diffractometer with an Atlas detector
Absorption correction: multi-scan (*CrysAlis PRO*; Agilent, 2010 ▶) *T*_min_ = 0.947, *T*_max_ = 1.000
20827 measured reflections
12691 independent reflections
8114 reflections with *I* > 2σ(*I*)
*R*_int_ = 0.036

#### Refinement

*R*[*F* ^2^ > 2σ(*F* ^2^)] = 0.056
*wR*(*F* ^2^) = 0.153
*S* = 1.02
12691 reflections
637 parameters
4 restraints
H-atom parameters constrained
Δρ_max_ = 0.29 e Å^−3^
Δρ_min_ = −0.34 e Å^−3^

### 

Data collection: *CrysAlis PRO* (Agilent, 2010 ▶); cell refinement: *CrysAlis PRO*; data reduction: *CrysAlis PRO*; program(s) used to solve structure: *SHELXS97* (Sheldrick 2008 ▶); program(s) used to refine structure: *SHELXL97* (Sheldrick, 2008 ▶; molecular graphics: *ORTEP-3 for Windows* (Farrugia, 1997 ▶); software used to prepare material for publication: *WinGX* (Farrugia, 1999 ▶).

## Supplementary Material

Crystal structure: contains datablock(s) I, global. DOI: 10.1107/S1600536811025943/ng5182sup1.cif

Structure factors: contains datablock(s) I. DOI: 10.1107/S1600536811025943/ng5182Isup2.hkl

Supplementary material file. DOI: 10.1107/S1600536811025943/ng5182Isup3.cml

Additional supplementary materials:  crystallographic information; 3D view; checkCIF report

## Figures and Tables

**Table 1:** Hydrogen-bond geometry (Å, °)

*D*—H⋯*A*	*D*—H	H⋯*A*	*D*⋯*A*	*D*—H⋯*A*
N1—H1⋯O1^i^	0.98	2.02	2.990 (2)	170
N1*B*—H1*B*⋯O21^ii^	0.90	2.15	3.016 (2)	163

Symmetry codes: (i) 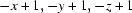; (ii) 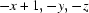.

**Fig. 1.**
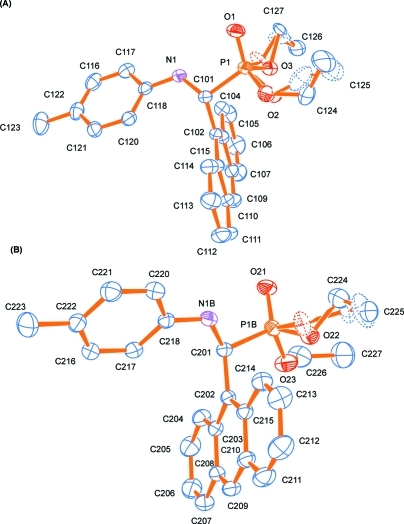
The asymmetric unit of title compound with the atom numbering scheme showing 50% probability displacement ellipsoids. H atoms have been omitted for clarity. The minor disorder component is shown as dasshed lines.

**Fig. 2.**
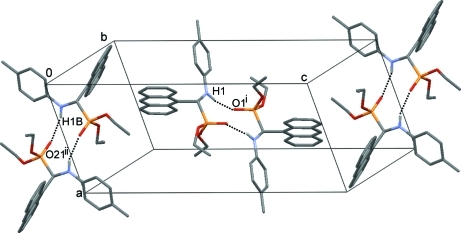
The packing arrangement of the molecules in the unit cell, showing the hydrogen-bonding interactions as dashed lines. [symmetry codes: (i) 1 - x, 1 - y, 1 - z; (ii) 1 - x, 1 - y, -z]

## supplementary crystallographic information

### Comment

Aminophosphonic acid derivatives constitute an important class of biologically active compounds with a great potential for medicinal and pharmaceutical applications. They are phosphorus analogues of natural alpha-aminocarboxylic acids - the bilding blocks of peptides and proteins Due to the close structural resemblance, aminophosphonates can mimic the aminoacids and can act as inhibitors and regulators of metabolic processes. Therefore they are extremely useful in the development of potential drugs against several metabolic disorders. Moreover, many of aminophosphonate derivatives exhibit antibacterial, antiviral and anticancer activity. The title compound has been synthesized and tested for cytotoxicity on Balb/c 3 T3 (clone31) cells, for *in vitro* antitumor activity using a panel of six human epithelial cancer cell lines and for genotoxicity and antiproliferative activity *in vivo*. Here we report its crystal structure.

The title compound (Fig. 1) possesses three distinct functional groups: anthracen, diethyl phosphonate and *p*-toluidine. It crystallizes with two independent molecules in the assymetric unit. The anthracen and toluidine moieties are nearly planar (with respective r.m.s. of 0.014/0.003 and 0.017/0.005Å for molecule A and B).

The interplanar angle beteween the anthracen and the toluidine is 82.92 (5) and 80.70 (5) °.

One can say that the diethyl phosphonate moiety is also positioned in the toluidine mean plane thus inferring a T-shape to the molecule.

In the crystal structure of the studied compound symmetrically equivalent molecules are linked together by N—H···O hydrogen bonds to form cyclic dimmers (Fig. 2).

Three of the four ethyl moieties present in the crystal structure of the title compound are disordered over two positions. The positional disorder on the O and C ethyl atoms was resolved by finding alternative positions from the difference Fourier map, and was subsequently refined over two positions. Where necessary bonds were constrained to distances of 1.54 (5) Å (P—O) and 1. 44 (5) Å (O—C and C—C). The occupancies of the component of the ethyl fragments starting with O2, O3 and O22 atoms are 0.737 (5), 0.755 (2) and 0.482 (3) respectively.

### Experimental

The studied compound has been obtained according to Kraicheva *et al.* 2011. Suitable crystals were obtained by slow evaporation from methanol/methylen chloride solution (1:1 *v*/*v*) at room temperature.

### Refinement

All H atoms bonded to C were placed in idealized positions (C—H_aromatic_ = 0.93 Å, *C*—H_methylen_ = 0.97 Å and *C*—H_methyl_ = 0.96 Å. The imino H atom was located from difference fourier map. All H atoms were constrained to ride on their parent atoms, with *U*_iso_(H) = 1.2*U*_eq_(C or N) and 1.5*U*_eq_(C_methyl_).

The positional disorder on the O and C ethyl atoms was resolved by finding alternative positions from the difference Fourier map, and was subsequently refined over two positions. Where necessary distance restraints (C—C= 1.52+/-0.05 Å, C—O=1.44+/-0.05 Å and P—O = 1.54+/-0.05 Å) were employed The occupancies of the component of the ethyl fragments starting with O2, O3 and O22 atoms are 0.748 (4), 0.768 (2) and 0.482 (3) respectively.

### Crystal data

**Table:**

C_26_H_28_NO_3_P	*F*(000) = 920
*M**_r_* = 433.46	*D*_x_ = 1.274 Mg m^−^^3^
Triclinic, *P*1	Melting point = 406–407 K
*a* = 9.5990 (3) Å	Mo *K*α radiation, λ = 0.7107 Å
*b* = 12.6386 (5) Å	Cell parameters from 6846 reflections
*c* = 20.2131 (7) Å	θ = 2.8–31.3°
α = 75.865 (3)°	µ = 0.15 mm^−^^1^
β = 76.780 (4)°	*T* = 290 K
γ = 74.927 (3)°	Prism, yellow
*V* = 2260.13 (14) Å^3^	0.31 × 0.18 × 0.15 mm
*Z* = 4	

### Data collection

**Table:**

Agilent SuperNova Dual diffractometer with an Atlas detector	12691 independent reflections
Radiation source: SuperNova (Mo) X-ray Source	8114 reflections with *I* > 2σ(*I*)
mirror	*R*_int_ = 0.036
Detector resolution: 10.3974 pixels mm^-1^	θ_max_ = 30.0°, θ_min_ = 2.8°
ω scans	*h* = −12→13
Absorption correction: multi-scan (*CrysAlis PRO*; Agilent, 2010)	*k* = −17→13
*T*_min_ = 0.947, *T*_max_ = 1.000	*l* = −28→28
20827 measured reflections	

### Refinement

**Table:**

Refinement on *F*^2^	Primary atom site location: structure-invariant direct methods
Least-squares matrix: full	Secondary atom site location: difference Fourier map
*R*[*F*^2^ > 2σ(*F*^2^)] = 0.056	Hydrogen site location: inferred from neighbouring sites
*wR*(*F*^2^) = 0.153	H-atom parameters constrained
*S* = 1.02	*w* = 1/[σ^2^(*F*_o_^2^) + (0.0574*P*)^2^ + 0.3871*P*] where *P* = (*F*_o_^2^ + 2*F*_c_^2^)/3
12691 reflections	(Δ/σ)_max_ = 0.001
637 parameters	Δρ_max_ = 0.29 e Å^−^^3^
4 restraints	Δρ_min_ = −0.34 e Å^−^^3^

### Special details

**Table:**

Geometry. All e.s.d.'s (except the e.s.d. in the dihedral angle between two l.s. planes) are estimated using the full covariance matrix. The cell e.s.d.'s are taken into account individually in the estimation of e.s.d.'s in distances, angles and torsion angles; correlations between e.s.d.'s in cell parameters are only used when they are defined by crystal symmetry. An approximate (isotropic) treatment of cell e.s.d.'s is used for estimating e.s.d.'s involving l.s. planes.
Refinement. Refinement of *F*^2^ against ALL reflections. The weighted *R*-factor *wR* and goodness of fit *S* are based on *F*^2^, conventional *R*-factors *R* are based on *F*, with *F* set to zero for negative *F*^2^. The threshold expression of *F*^2^ > σ(*F*^2^) is used only for calculating *R*-factors(gt) *etc*. and is not relevant to the choice of reflections for refinement. *R*-factors based on *F*^2^ are statistically about twice as large as those based on *F*, and *R*- factors based on ALL data will be even larger.

### Fractional atomic coordinates and isotropic or equivalent isotropic displacement parameters (Å^2^)

**Table:**

	*x*	*y*	*z*	*U*_iso_*/*U*_eq_	Occ. (<1)
C101	0.32852 (19)	0.42066 (15)	0.42195 (9)	0.0383 (4)	
H101	0.2516	0.3873	0.4545	0.046*	
C102	0.32061 (18)	0.40400 (14)	0.35010 (9)	0.0360 (4)	
C103	0.38352 (19)	0.46855 (15)	0.28834 (9)	0.0399 (4)	
C104	0.4604 (2)	0.55260 (17)	0.28607 (11)	0.0502 (5)	
H104	0.4707	0.5669	0.3275	0.060*	
C105	0.5185 (3)	0.6119 (2)	0.22562 (12)	0.0632 (6)	
H105	0.5685	0.6654	0.2264	0.076*	
C106	0.5050 (3)	0.5943 (2)	0.16170 (12)	0.0741 (7)	
H106	0.5444	0.6367	0.1206	0.089*	
C107	0.4352 (3)	0.5163 (2)	0.16009 (11)	0.0675 (7)	
H107	0.4271	0.5048	0.1175	0.081*	
C108	0.3723 (2)	0.45006 (18)	0.22268 (9)	0.0470 (5)	
C109	0.3007 (2)	0.36943 (19)	0.22033 (10)	0.0523 (5)	
H109	0.2944	0.3582	0.1774	0.063*	
C110	0.2384 (2)	0.30518 (16)	0.27977 (10)	0.0440 (4)	
C111	0.1637 (3)	0.22403 (19)	0.27558 (12)	0.0587 (6)	
H111	0.1601	0.2128	0.2323	0.070*	
C113	0.1044 (3)	0.17947 (19)	0.39868 (13)	0.0623 (6)	
H113	0.0585	0.1377	0.4382	0.075*	
C114	0.1760 (2)	0.25470 (17)	0.40555 (10)	0.0502 (5)	
H114	0.1789	0.2625	0.4497	0.060*	
C115	0.24696 (19)	0.32237 (15)	0.34655 (9)	0.0386 (4)	
C116	0.0284 (3)	0.79981 (17)	0.40743 (11)	0.0584 (6)	
H116	0.0247	0.8750	0.4046	0.070*	
C117	0.1572 (2)	0.72306 (17)	0.41614 (10)	0.0515 (5)	
H117	0.2389	0.7475	0.4187	0.062*	
C118	0.1675 (2)	0.60930 (15)	0.42123 (8)	0.0402 (4)	
C120	0.0428 (2)	0.57649 (16)	0.41685 (9)	0.0443 (4)	
H120	0.0457	0.5014	0.4198	0.053*	
C121	−0.0860 (2)	0.65542 (18)	0.40804 (10)	0.0493 (5)	
H121	−0.1683	0.6316	0.4056	0.059*	
C122	−0.0961 (2)	0.76765 (18)	0.40273 (10)	0.0534 (5)	
C123	−0.2378 (3)	0.8513 (2)	0.39294 (14)	0.0830 (8)	
H23A	−0.2910	0.8245	0.3680	0.124*	
H23B	−0.2167	0.9216	0.3672	0.124*	
H23C	−0.2958	0.8608	0.4374	0.124*	
C126	0.8357 (3)	0.4455 (2)	0.32847 (12)	0.0691 (7)	
H26A	0.9106	0.4784	0.3343	0.104*	
H26B	0.7788	0.4970	0.2961	0.104*	
H26C	0.8804	0.3779	0.3112	0.104*	
C201	0.19612 (18)	0.10024 (15)	0.06409 (8)	0.0368 (4)	
H201	0.1381	0.0438	0.0711	0.044*	
C202	0.09303 (18)	0.19745 (14)	0.09577 (8)	0.0350 (4)	
C203	−0.02950 (19)	0.17438 (15)	0.14672 (8)	0.0381 (4)	
C204	−0.0612 (2)	0.06593 (18)	0.17192 (10)	0.0501 (5)	
H204	0.0024	0.0055	0.1549	0.060*	
C205	−0.1815 (3)	0.0481 (2)	0.22005 (11)	0.0650 (6)	
H205	−0.1992	−0.0236	0.2348	0.078*	
C206	−0.2798 (3)	0.1369 (3)	0.24797 (12)	0.0695 (7)	
H206	−0.3616	0.1234	0.2810	0.083*	
C207	−0.2557 (2)	0.2408 (2)	0.22694 (11)	0.0616 (6)	
H207	−0.3208	0.2987	0.2460	0.074*	
C208	−0.1315 (2)	0.26435 (17)	0.17570 (9)	0.0438 (4)	
C209	−0.1076 (2)	0.37117 (17)	0.15378 (10)	0.0501 (5)	
H209	−0.1754	0.4290	0.1719	0.060*	
C210	0.0136 (2)	0.39572 (15)	0.10583 (9)	0.0438 (4)	
C211	0.0369 (3)	0.50719 (17)	0.08596 (12)	0.0588 (6)	
H211	−0.0311	0.5636	0.1053	0.071*	
C212	0.1541 (3)	0.53224 (19)	0.04032 (13)	0.0661 (6)	
H212	0.1671	0.6053	0.0280	0.079*	
C213	0.2572 (3)	0.44751 (18)	0.01113 (11)	0.0601 (6)	
H213	0.3391	0.4653	−0.0202	0.072*	
C214	0.2408 (2)	0.34046 (17)	0.02739 (10)	0.0481 (4)	
H214	0.3113	0.2867	0.0066	0.058*	
C215	0.11737 (19)	0.30786 (14)	0.07585 (9)	0.0374 (4)	
C216	−0.0702 (2)	0.19826 (17)	−0.09719 (10)	0.0476 (5)	
H216	−0.1698	0.1986	−0.0865	0.057*	
C217	0.0126 (2)	0.15950 (16)	−0.04435 (9)	0.0438 (4)	
H217	−0.0318	0.1344	0.0008	0.053*	
C218	0.16218 (19)	0.15833 (14)	−0.05918 (9)	0.0387 (4)	
C220	0.2244 (2)	0.19460 (16)	−0.12763 (10)	0.0454 (4)	
H220	0.3244	0.1931	−0.1389	0.055*	
C221	0.1408 (2)	0.23251 (17)	−0.17861 (10)	0.0517 (5)	
H221	0.1855	0.2565	−0.2238	0.062*	
C222	−0.0089 (2)	0.23628 (17)	−0.16500 (10)	0.0476 (5)	
C223	−0.0985 (3)	0.2816 (3)	−0.22198 (12)	0.0806 (8)	
H23D	−0.1994	0.2799	−0.2031	0.121*	
H23E	−0.0632	0.2365	−0.2568	0.121*	
H23F	−0.0901	0.3572	−0.2423	0.121*	
C226	0.3190 (3)	−0.0812 (2)	0.23724 (13)	0.0782 (7)	
H26D	0.3547	−0.1457	0.2153	0.094*	
H26E	0.2307	−0.0921	0.2703	0.094*	
C227	0.4284 (4)	−0.0743 (3)	0.27378 (16)	0.1135 (12)	
H27D	0.5182	−0.0687	0.2418	0.170*	
H27E	0.4449	−0.1402	0.3091	0.170*	
H27F	0.3946	−0.0096	0.2947	0.170*	
C112	0.0984 (3)	0.1634 (2)	0.33272 (14)	0.0661 (6)	
H112	0.0495	0.1112	0.3289	0.079*	
N1	0.29837 (18)	0.53514 (13)	0.43061 (8)	0.0485 (4)	
H1	0.3674	0.5597	0.4493	0.058*	
N1B	0.25112 (17)	0.12429 (14)	−0.00954 (7)	0.0459 (4)	
H1B	0.3479	0.1026	−0.0232	0.055*	
O1	0.50897 (16)	0.36126 (12)	0.51906 (7)	0.0542 (4)	
O2	0.4889 (7)	0.2224 (6)	0.4522 (4)	0.0535 (11)	0.748 (15)
C124	0.6130 (7)	0.1334 (4)	0.4410 (5)	0.0776 (17)	0.748 (15)
H24A	0.6826	0.1609	0.4017	0.093*	0.748 (15)
H24B	0.5814	0.0746	0.4291	0.093*	0.748 (15)
C125	0.6876 (13)	0.0857 (6)	0.5009 (6)	0.128 (4)	0.748 (15)
H25A	0.7685	0.0259	0.4899	0.193*	0.748 (15)
H25B	0.6199	0.0575	0.5400	0.193*	0.748 (15)
H25C	0.7231	0.1425	0.5119	0.193*	0.748 (15)
O2B	0.531 (3)	0.229 (2)	0.4318 (13)	0.078 (6)	0.252 (15)
C324	0.592 (2)	0.1266 (13)	0.4840 (15)	0.094 (6)	0.252 (15)
H32A	0.5727	0.1416	0.5304	0.113*	0.252 (15)
H32B	0.5553	0.0617	0.4847	0.113*	0.252 (15)
C325	0.742 (2)	0.1148 (19)	0.4540 (14)	0.110 (7)	0.252 (15)
H32C	0.7990	0.0533	0.4817	0.164*	0.252 (15)
H32D	0.7730	0.1822	0.4517	0.164*	0.252 (15)
H32E	0.7567	0.1007	0.4080	0.164*	0.252 (15)
O23	0.28356 (18)	0.01628 (13)	0.18572 (7)	0.0675 (5)	
O3	0.6276 (8)	0.3689 (6)	0.3922 (3)	0.0490 (12)	0.77 (4)
C127	0.7412 (11)	0.4202 (8)	0.3947 (6)	0.077 (3)	0.77 (4)
H12D	0.6972	0.4889	0.4118	0.092*	0.77 (4)
H12E	0.8006	0.3710	0.4276	0.092*	0.77 (4)
O3B	0.619 (3)	0.404 (5)	0.3910 (13)	0.079 (7)	0.23 (4)
C273	0.754 (3)	0.4200 (16)	0.4067 (15)	0.046 (4)	0.23 (4)
H27A	0.8060	0.3530	0.4336	0.055*	0.23 (4)
H27B	0.7363	0.4825	0.4296	0.055*	0.23 (4)
O21	0.43711 (15)	−0.07165 (12)	0.08495 (7)	0.0539 (4)	
O22	0.4284 (11)	0.1262 (8)	0.1064 (5)	0.0494 (15)	0.482 (19)
C224	0.5885 (18)	0.1236 (15)	0.0752 (7)	0.065 (3)	0.482 (19)
H22D	0.6493	0.0666	0.1045	0.078*	0.482 (19)
H22E	0.6092	0.1035	0.0300	0.078*	0.482 (19)
C225	0.6267 (16)	0.2323 (10)	0.0677 (6)	0.056 (3)	0.482 (19)
H22A	0.7314	0.2229	0.0582	0.084*	0.482 (19)
H22B	0.5877	0.2600	0.1099	0.084*	0.482 (19)
H22C	0.5859	0.2845	0.0302	0.084*	0.482 (19)
O22B	0.4432 (11)	0.1264 (8)	0.0802 (6)	0.0587 (18)	0.518 (19)
C229	0.5762 (16)	0.1197 (14)	0.1033 (8)	0.070 (3)	0.518 (19)
H22F	0.6512	0.0561	0.0911	0.085*	0.518 (19)
H22G	0.5600	0.1150	0.1530	0.085*	0.518 (19)
C425	0.616 (2)	0.2254 (15)	0.0652 (9)	0.112 (6)	0.518 (19)
H42A	0.6900	0.2392	0.0847	0.168*	0.518 (19)
H42B	0.5306	0.2852	0.0685	0.168*	0.518 (19)
H42C	0.6523	0.2210	0.0174	0.168*	0.518 (19)
P1	0.49980 (6)	0.34524 (4)	0.45120 (3)	0.04332 (14)	
P1B	0.35236 (5)	0.03141 (4)	0.10715 (3)	0.04175 (13)	

### Atomic displacement parameters (Å^2^)

**Table:**

	*U*^11^	*U*^22^	*U*^33^	*U*^12^	*U*^13^	*U*^23^
C101	0.0405 (9)	0.0401 (9)	0.0382 (9)	−0.0057 (8)	−0.0127 (8)	−0.0131 (8)
C102	0.0363 (9)	0.0367 (9)	0.0370 (9)	−0.0004 (7)	−0.0135 (7)	−0.0123 (7)
C103	0.0360 (9)	0.0423 (10)	0.0399 (9)	0.0004 (8)	−0.0097 (7)	−0.0117 (8)
C104	0.0481 (11)	0.0518 (12)	0.0512 (11)	−0.0111 (9)	−0.0118 (9)	−0.0077 (9)
C105	0.0557 (13)	0.0623 (14)	0.0660 (14)	−0.0174 (11)	−0.0052 (11)	−0.0022 (12)
C106	0.0728 (16)	0.0869 (19)	0.0522 (13)	−0.0262 (15)	0.0079 (12)	−0.0026 (13)
C107	0.0696 (15)	0.0884 (18)	0.0393 (11)	−0.0176 (14)	0.0011 (10)	−0.0125 (11)
C108	0.0440 (10)	0.0573 (12)	0.0369 (9)	−0.0030 (9)	−0.0062 (8)	−0.0131 (9)
C109	0.0521 (11)	0.0684 (14)	0.0391 (10)	−0.0034 (10)	−0.0090 (9)	−0.0250 (10)
C110	0.0419 (10)	0.0476 (11)	0.0473 (10)	0.0007 (8)	−0.0145 (8)	−0.0233 (9)
C111	0.0644 (14)	0.0589 (13)	0.0661 (14)	−0.0070 (11)	−0.0234 (12)	−0.0328 (12)
C113	0.0703 (15)	0.0520 (13)	0.0707 (14)	−0.0226 (11)	−0.0216 (12)	−0.0047 (11)
C114	0.0591 (12)	0.0472 (11)	0.0494 (11)	−0.0145 (10)	−0.0174 (10)	−0.0085 (9)
C115	0.0399 (9)	0.0378 (9)	0.0407 (9)	−0.0023 (7)	−0.0141 (8)	−0.0125 (8)
C116	0.0786 (16)	0.0376 (11)	0.0523 (12)	−0.0035 (11)	−0.0083 (11)	−0.0094 (9)
C117	0.0605 (13)	0.0469 (11)	0.0505 (11)	−0.0125 (10)	−0.0100 (10)	−0.0149 (9)
C118	0.0478 (10)	0.0427 (10)	0.0307 (8)	−0.0055 (8)	−0.0086 (8)	−0.0116 (7)
C120	0.0503 (11)	0.0415 (10)	0.0420 (9)	−0.0068 (8)	−0.0102 (8)	−0.0114 (8)
C121	0.0454 (11)	0.0572 (12)	0.0421 (10)	−0.0039 (9)	−0.0081 (9)	−0.0113 (9)
C122	0.0591 (13)	0.0509 (12)	0.0381 (10)	0.0055 (10)	−0.0060 (9)	−0.0070 (9)
C123	0.0782 (18)	0.0672 (16)	0.0792 (17)	0.0191 (14)	−0.0132 (14)	−0.0074 (13)
C126	0.0499 (13)	0.0983 (19)	0.0649 (14)	−0.0136 (13)	−0.0065 (11)	−0.0329 (14)
C201	0.0352 (8)	0.0374 (9)	0.0378 (9)	−0.0014 (7)	−0.0071 (7)	−0.0136 (7)
C202	0.0344 (8)	0.0366 (9)	0.0337 (8)	0.0003 (7)	−0.0105 (7)	−0.0107 (7)
C203	0.0379 (9)	0.0449 (10)	0.0325 (8)	−0.0054 (8)	−0.0085 (7)	−0.0115 (7)
C204	0.0535 (12)	0.0527 (12)	0.0440 (10)	−0.0131 (10)	−0.0016 (9)	−0.0138 (9)
C205	0.0701 (15)	0.0769 (16)	0.0514 (12)	−0.0353 (13)	0.0045 (11)	−0.0123 (12)
C206	0.0564 (14)	0.104 (2)	0.0535 (13)	−0.0337 (14)	0.0101 (11)	−0.0255 (13)
C207	0.0449 (11)	0.0882 (18)	0.0534 (12)	−0.0068 (12)	0.0011 (10)	−0.0340 (12)
C208	0.0363 (9)	0.0571 (12)	0.0389 (9)	−0.0010 (8)	−0.0075 (8)	−0.0198 (9)
C209	0.0481 (11)	0.0520 (12)	0.0499 (11)	0.0082 (9)	−0.0126 (9)	−0.0261 (10)
C210	0.0502 (11)	0.0392 (10)	0.0421 (9)	0.0027 (8)	−0.0155 (9)	−0.0145 (8)
C211	0.0740 (15)	0.0388 (11)	0.0640 (13)	0.0027 (10)	−0.0198 (12)	−0.0198 (10)
C212	0.0927 (19)	0.0393 (11)	0.0672 (14)	−0.0149 (12)	−0.0171 (14)	−0.0086 (11)
C213	0.0709 (15)	0.0497 (12)	0.0576 (13)	−0.0204 (11)	−0.0046 (11)	−0.0053 (10)
C214	0.0509 (11)	0.0434 (11)	0.0479 (10)	−0.0080 (9)	−0.0058 (9)	−0.0100 (9)
C215	0.0400 (9)	0.0353 (9)	0.0370 (9)	−0.0011 (7)	−0.0114 (7)	−0.0106 (7)
C216	0.0389 (10)	0.0560 (12)	0.0498 (11)	−0.0021 (9)	−0.0097 (9)	−0.0209 (9)
C217	0.0428 (10)	0.0495 (11)	0.0380 (9)	−0.0053 (8)	−0.0042 (8)	−0.0146 (8)
C218	0.0395 (9)	0.0357 (9)	0.0416 (9)	0.0005 (7)	−0.0099 (8)	−0.0157 (8)
C220	0.0413 (10)	0.0465 (11)	0.0457 (10)	−0.0070 (8)	−0.0034 (8)	−0.0106 (9)
C221	0.0546 (12)	0.0531 (12)	0.0421 (10)	−0.0073 (10)	−0.0068 (9)	−0.0060 (9)
C222	0.0505 (11)	0.0495 (11)	0.0442 (10)	0.0002 (9)	−0.0160 (9)	−0.0162 (9)
C223	0.0679 (16)	0.111 (2)	0.0574 (14)	0.0031 (15)	−0.0263 (13)	−0.0154 (14)
C226	0.0806 (18)	0.0803 (18)	0.0590 (14)	−0.0117 (15)	−0.0067 (13)	0.0021 (13)
C227	0.109 (3)	0.142 (3)	0.084 (2)	0.012 (2)	−0.055 (2)	−0.016 (2)
C112	0.0738 (16)	0.0536 (13)	0.0876 (17)	−0.0201 (12)	−0.0275 (14)	−0.0258 (13)
N1	0.0481 (9)	0.0455 (9)	0.0609 (10)	−0.0010 (7)	−0.0225 (8)	−0.0251 (8)
N1B	0.0336 (8)	0.0616 (10)	0.0390 (8)	0.0027 (7)	−0.0053 (6)	−0.0184 (7)
O1	0.0599 (9)	0.0638 (9)	0.0471 (8)	−0.0125 (7)	−0.0248 (7)	−0.0124 (7)
O2	0.058 (2)	0.0393 (16)	0.067 (2)	−0.0059 (19)	−0.0242 (17)	−0.0098 (18)
C124	0.087 (3)	0.049 (2)	0.096 (4)	0.007 (2)	−0.021 (3)	−0.032 (3)
C125	0.157 (8)	0.067 (3)	0.177 (8)	0.031 (4)	−0.121 (7)	−0.027 (4)
O2B	0.085 (13)	0.058 (9)	0.104 (14)	0.029 (8)	−0.062 (10)	−0.045 (9)
C324	0.127 (14)	0.052 (8)	0.106 (15)	−0.030 (9)	0.008 (13)	−0.033 (10)
C325	0.100 (14)	0.095 (12)	0.133 (17)	−0.013 (9)	−0.047 (13)	−0.004 (12)
O23	0.0779 (11)	0.0682 (10)	0.0406 (8)	0.0215 (8)	−0.0197 (7)	−0.0129 (7)
O3	0.0478 (18)	0.057 (2)	0.048 (2)	−0.0098 (16)	−0.0123 (15)	−0.0194 (19)
C127	0.072 (4)	0.134 (6)	0.051 (3)	−0.054 (4)	−0.012 (3)	−0.032 (3)
O3B	0.039 (6)	0.11 (2)	0.073 (8)	−0.017 (10)	−0.027 (6)	0.017 (9)
C273	0.039 (6)	0.044 (9)	0.064 (11)	−0.006 (6)	−0.028 (7)	−0.014 (6)
O21	0.0446 (7)	0.0513 (8)	0.0595 (8)	0.0090 (6)	−0.0094 (7)	−0.0197 (7)
O22	0.045 (2)	0.046 (2)	0.058 (4)	−0.0084 (16)	−0.006 (3)	−0.016 (3)
C224	0.050 (4)	0.066 (5)	0.077 (6)	−0.007 (3)	−0.007 (5)	−0.023 (6)
C225	0.048 (5)	0.061 (5)	0.066 (6)	−0.018 (4)	−0.008 (4)	−0.021 (4)
O22B	0.046 (3)	0.062 (3)	0.072 (4)	−0.016 (2)	−0.025 (4)	−0.001 (4)
C229	0.053 (6)	0.070 (4)	0.101 (8)	−0.014 (4)	−0.042 (7)	−0.016 (7)
C425	0.067 (6)	0.156 (12)	0.124 (10)	−0.067 (7)	−0.022 (6)	0.004 (9)
P1	0.0467 (3)	0.0427 (3)	0.0453 (3)	−0.0046 (2)	−0.0201 (2)	−0.0117 (2)
P1B	0.0369 (2)	0.0411 (3)	0.0455 (3)	0.0009 (2)	−0.0121 (2)	−0.0107 (2)

### Geometric parameters (Å, °)

**Table:**

C101—N1	1.446 (2)	C213—C214	1.354 (3)
C101—C102	1.539 (2)	C213—H213	0.9300
C101—P1	1.8208 (17)	C214—C215	1.435 (3)
C101—H101	0.9800	C214—H214	0.9300
C102—C103	1.415 (2)	C216—C222	1.383 (3)
C102—C115	1.417 (2)	C216—C217	1.394 (3)
C103—C104	1.429 (3)	C216—H216	0.9300
C103—C108	1.434 (2)	C217—C218	1.395 (3)
C104—C105	1.351 (3)	C217—H217	0.9300
C104—H104	0.9300	C218—N1B	1.382 (2)
C105—C106	1.403 (3)	C218—C220	1.391 (2)
C105—H105	0.9300	C220—C221	1.369 (3)
C106—C107	1.338 (3)	C220—H220	0.9300
C106—H106	0.9300	C221—C222	1.390 (3)
C107—C108	1.436 (3)	C221—H221	0.9300
C107—H107	0.9300	C222—C223	1.506 (3)
C108—C109	1.384 (3)	C223—H23D	0.9600
C109—C110	1.381 (3)	C223—H23E	0.9600
C109—H109	0.9300	C223—H23F	0.9600
C110—C111	1.423 (3)	C226—O23	1.426 (3)
C110—C115	1.442 (2)	C226—C227	1.446 (4)
C111—C112	1.342 (3)	C226—H26D	0.9700
C111—H111	0.9300	C226—H26E	0.9700
C113—C114	1.357 (3)	C227—H27D	0.9600
C113—C112	1.413 (3)	C227—H27E	0.9600
C113—H113	0.9300	C227—H27F	0.9600
C114—C115	1.432 (3)	C112—H112	0.9300
C114—H114	0.9300	N1—H1	0.9784
C116—C117	1.376 (3)	N1B—H1B	0.8955
C116—C122	1.387 (3)	O1—P1	1.4596 (13)
C116—H116	0.9300	O2—C124	1.428 (9)
C117—C118	1.395 (3)	O2—P1	1.577 (8)
C117—H117	0.9300	C124—C125	1.468 (12)
C118—N1	1.379 (2)	C124—H24A	0.9700
C118—C120	1.392 (3)	C124—H24B	0.9700
C120—C121	1.389 (3)	C125—H25A	0.9600
C120—H120	0.9300	C125—H25B	0.9600
C121—C122	1.376 (3)	C125—H25C	0.9600
C121—H121	0.9300	O2B—P1	1.55 (2)
C122—C123	1.510 (3)	O2B—C324	1.54 (3)
C123—H23A	0.9600	C324—C325	1.41 (3)
C123—H23B	0.9600	C324—H32A	0.9700
C123—H23C	0.9600	C324—H32B	0.9700
C126—C127	1.451 (10)	C325—H32C	0.9600
C126—C273	1.59 (3)	C325—H32D	0.9600
C126—H26A	0.9600	C325—H32E	0.9600
C126—H26B	0.9600	O23—P1B	1.5598 (15)
C126—H26C	0.9600	O3—C127	1.420 (11)
C201—N1B	1.445 (2)	O3—P1	1.537 (7)
C201—C202	1.537 (2)	C127—H12D	0.9700
C201—P1B	1.8200 (17)	C127—H12E	0.9700
C201—H201	0.9800	O3B—C273	1.48 (3)
C202—C203	1.417 (2)	O3B—P1	1.64 (3)
C202—C215	1.420 (2)	C273—H27A	0.9700
C203—C204	1.427 (3)	C273—H27B	0.9700
C203—C208	1.442 (2)	O21—P1B	1.4584 (14)
C204—C205	1.358 (3)	O22—C224	1.517 (18)
C204—H204	0.9300	O22—P1B	1.553 (9)
C205—C206	1.408 (3)	C224—C225	1.48 (2)
C205—H205	0.9300	C224—H22D	0.9700
C206—C207	1.342 (3)	C224—H22E	0.9700
C206—H206	0.9300	C225—H22A	0.9600
C207—C208	1.432 (3)	C225—H22B	0.9600
C207—H207	0.9300	C225—H22C	0.9600
C208—C209	1.376 (3)	O22B—C229	1.432 (16)
C209—C210	1.383 (3)	O22B—P1B	1.583 (9)
C209—H209	0.9300	C229—C425	1.47 (2)
C210—C211	1.429 (3)	C229—H22F	0.9700
C210—C215	1.439 (2)	C229—H22G	0.9700
C211—C212	1.336 (3)	C425—H42A	0.9600
C211—H211	0.9300	C425—H42B	0.9600
C212—C213	1.402 (3)	C425—H42C	0.9600
C212—H212	0.9300		
			
N1—C101—C102	116.10 (14)	C216—C217—H217	120.0
N1—C101—P1	109.21 (11)	C218—C217—H217	120.0
C102—C101—P1	113.18 (12)	N1B—C218—C220	118.43 (17)
N1—C101—H101	105.8	N1B—C218—C217	123.58 (17)
C102—C101—H101	105.8	C220—C218—C217	117.99 (17)
P1—C101—H101	105.8	C221—C220—C218	120.97 (18)
C103—C102—C115	119.87 (15)	C221—C220—H220	119.5
C103—C102—C101	121.64 (15)	C218—C220—H220	119.5
C115—C102—C101	118.49 (15)	C220—C221—C222	122.15 (19)
C102—C103—C104	124.40 (16)	C220—C221—H221	118.9
C102—C103—C108	119.39 (17)	C222—C221—H221	118.9
C104—C103—C108	116.21 (17)	C216—C222—C221	116.85 (17)
C105—C104—C103	122.1 (2)	C216—C222—C223	122.4 (2)
C105—C104—H104	119.0	C221—C222—C223	120.77 (19)
C103—C104—H104	119.0	C222—C223—H23D	109.5
C104—C105—C106	121.2 (2)	C222—C223—H23E	109.5
C104—C105—H105	119.4	H23D—C223—H23E	109.5
C106—C105—H105	119.4	C222—C223—H23F	109.5
C107—C106—C105	119.8 (2)	H23D—C223—H23F	109.5
C107—C106—H106	120.1	H23E—C223—H23F	109.5
C105—C106—H106	120.1	O23—C226—C227	112.8 (3)
C106—C107—C108	121.4 (2)	O23—C226—H26D	109.0
C106—C107—H107	119.3	C227—C226—H26D	109.0
C108—C107—H107	119.3	O23—C226—H26E	109.0
C109—C108—C103	119.85 (18)	C227—C226—H26E	109.0
C109—C108—C107	120.88 (18)	H26D—C226—H26E	107.8
C103—C108—C107	119.3 (2)	C226—C227—H27D	109.5
C110—C109—C108	121.99 (17)	C226—C227—H27E	109.5
C110—C109—H109	119.0	H27D—C227—H27E	109.5
C108—C109—H109	119.0	C226—C227—H27F	109.5
C109—C110—C111	120.60 (18)	H27D—C227—H27F	109.5
C109—C110—C115	119.46 (17)	H27E—C227—H27F	109.5
C111—C110—C115	119.92 (19)	C111—C112—C113	119.6 (2)
C112—C111—C110	121.45 (19)	C111—C112—H112	120.2
C112—C111—H111	119.3	C113—C112—H112	120.2
C110—C111—H111	119.3	C118—N1—C101	122.73 (15)
C114—C113—C112	121.3 (2)	C118—N1—H1	117.3
C114—C113—H113	119.4	C101—N1—H1	119.6
C112—C113—H113	119.4	C218—N1B—C201	123.52 (15)
C113—C114—C115	121.76 (19)	C218—N1B—H1B	118.4
C113—C114—H114	119.1	C201—N1B—H1B	116.9
C115—C114—H114	119.1	C124—O2—P1	123.9 (5)
C102—C115—C114	124.57 (16)	O2—C124—C125	114.0 (9)
C102—C115—C110	119.44 (17)	O2—C124—H24A	108.7
C114—C115—C110	115.99 (17)	C125—C124—H24A	108.7
C117—C116—C122	121.6 (2)	O2—C124—H24B	108.7
C117—C116—H116	119.2	C125—C124—H24B	108.7
C122—C116—H116	119.2	H24A—C124—H24B	107.6
C116—C117—C118	121.2 (2)	C124—C125—H25A	109.5
C116—C117—H117	119.4	C124—C125—H25B	109.5
C118—C117—H117	119.4	H25A—C125—H25B	109.5
N1—C118—C120	123.23 (17)	C124—C125—H25C	109.5
N1—C118—C117	119.26 (17)	H25A—C125—H25C	109.5
C120—C118—C117	117.51 (18)	H25B—C125—H25C	109.5
C121—C120—C118	120.30 (18)	P1—O2B—C324	117.5 (18)
C121—C120—H120	119.8	C325—C324—O2B	98 (3)
C118—C120—H120	119.8	C325—C324—H32A	112.1
C122—C121—C120	122.2 (2)	O2B—C324—H32A	112.1
C122—C121—H121	118.9	C325—C324—H32B	112.1
C120—C121—H121	118.9	O2B—C324—H32B	112.1
C121—C122—C116	117.21 (19)	H32A—C324—H32B	109.7
C121—C122—C123	120.8 (2)	C324—C325—H32C	109.5
C116—C122—C123	122.0 (2)	C324—C325—H32D	109.5
C122—C123—H23A	109.5	H32C—C325—H32D	109.5
C122—C123—H23B	109.5	C324—C325—H32E	109.5
H23A—C123—H23B	109.5	H32C—C325—H32E	109.5
C122—C123—H23C	109.5	H32D—C325—H32E	109.5
H23A—C123—H23C	109.5	C226—O23—P1B	125.73 (15)
H23B—C123—H23C	109.5	C127—O3—P1	126.4 (6)
C127—C126—H26A	109.5	C126—C127—O3	114.1 (8)
C273—C126—H26A	100.0	C126—C127—H12D	108.7
C127—C126—H26B	109.5	O3—C127—H12D	108.7
C273—C126—H26B	116.5	C126—C127—H12E	108.7
H26A—C126—H26B	109.5	O3—C127—H12E	108.7
C127—C126—H26C	109.5	H12D—C127—H12E	107.6
C273—C126—H26C	111.4	C273—O3B—P1	121.8 (19)
H26A—C126—H26C	109.5	O3B—C273—C126	96.5 (18)
H26B—C126—H26C	109.5	O3B—C273—H27A	112.5
N1B—C201—C202	116.66 (14)	C126—C273—H27A	112.5
N1B—C201—P1B	107.55 (11)	O3B—C273—H27B	112.5
C202—C201—P1B	114.17 (11)	C126—C273—H27B	112.5
N1B—C201—H201	105.9	H27A—C273—H27B	110.0
C202—C201—H201	105.9	C224—O22—P1B	122.6 (9)
P1B—C201—H201	105.9	C225—C224—O22	112.1 (12)
C203—C202—C215	119.74 (15)	C225—C224—H22D	109.2
C203—C202—C201	118.02 (15)	O22—C224—H22D	109.2
C215—C202—C201	122.24 (15)	C225—C224—H22E	109.2
C202—C203—C204	124.16 (17)	O22—C224—H22E	109.2
C202—C203—C208	119.50 (17)	H22D—C224—H22E	107.9
C204—C203—C208	116.33 (17)	C224—C225—H22A	109.5
C205—C204—C203	122.1 (2)	C224—C225—H22B	109.5
C205—C204—H204	119.0	H22A—C225—H22B	109.5
C203—C204—H204	119.0	C224—C225—H22C	109.5
C204—C205—C206	120.9 (2)	H22A—C225—H22C	109.5
C204—C205—H205	119.6	H22B—C225—H22C	109.5
C206—C205—H205	119.6	C229—O22B—P1B	122.6 (9)
C207—C206—C205	120.1 (2)	O22B—C229—C425	101.9 (13)
C207—C206—H206	120.0	O22B—C229—H22F	111.4
C205—C206—H206	120.0	C425—C229—H22F	111.4
C206—C207—C208	121.3 (2)	O22B—C229—H22G	111.4
C206—C207—H207	119.4	C425—C229—H22G	111.4
C208—C207—H207	119.4	H22F—C229—H22G	109.3
C209—C208—C207	121.20 (19)	C229—C425—H42A	109.5
C209—C208—C203	119.44 (17)	C229—C425—H42B	109.5
C207—C208—C203	119.36 (19)	H42A—C425—H42B	109.5
C208—C209—C210	122.40 (17)	C229—C425—H42C	109.5
C208—C209—H209	118.8	H42A—C425—H42C	109.5
C210—C209—H209	118.8	H42B—C425—H42C	109.5
C209—C210—C211	120.74 (18)	O1—P1—O3	116.2 (2)
C209—C210—C215	119.54 (18)	O1—P1—O2B	123.7 (8)
C211—C210—C215	119.71 (19)	O3—P1—O2B	86.3 (10)
C212—C211—C210	121.6 (2)	O1—P1—O2	112.7 (3)
C212—C211—H211	119.2	O3—P1—O2	105.1 (3)
C210—C211—H211	119.2	O1—P1—O3B	109.9 (14)
C211—C212—C213	119.5 (2)	O2B—P1—O3B	101 (2)
C211—C212—H212	120.2	O2—P1—O3B	120 (2)
C213—C212—H212	120.2	O1—P1—C101	113.62 (8)
C214—C213—C212	121.8 (2)	O3—P1—C101	108.5 (3)
C214—C213—H213	119.1	O2B—P1—C101	104.8 (9)
C212—C213—H213	119.1	O2—P1—C101	99.0 (3)
C213—C214—C215	121.6 (2)	O3B—P1—C101	101.0 (11)
C213—C214—H214	119.2	O21—P1B—O22	120.8 (3)
C215—C214—H214	119.2	O21—P1B—O23	114.37 (8)
C202—C215—C214	124.89 (16)	O22—P1B—O23	95.8 (3)
C202—C215—C210	119.31 (16)	O21—P1B—O22B	110.3 (4)
C214—C215—C210	115.81 (17)	O23—P1B—O22B	114.6 (4)
C222—C216—C217	122.05 (18)	O21—P1B—C201	114.42 (8)
C222—C216—H216	119.0	O22—P1B—C201	105.2 (4)
C217—C216—H216	119.0	O23—P1B—C201	103.62 (8)
C216—C217—C218	119.96 (17)	O22B—P1B—C201	98.3 (3)
			
N1—C101—C102—C103	44.0 (2)	C209—C210—C215—C214	−179.10 (16)
P1—C101—C102—C103	−83.48 (18)	C211—C210—C215—C214	0.7 (2)
N1—C101—C102—C115	−135.43 (17)	C222—C216—C217—C218	0.0 (3)
P1—C101—C102—C115	97.10 (16)	C216—C217—C218—N1B	178.08 (17)
C115—C102—C103—C104	−179.81 (17)	C216—C217—C218—C220	−1.2 (3)
C101—C102—C103—C104	0.8 (3)	N1B—C218—C220—C221	−177.97 (18)
C115—C102—C103—C108	−0.2 (2)	C217—C218—C220—C221	1.4 (3)
C101—C102—C103—C108	−179.66 (15)	C218—C220—C221—C222	−0.3 (3)
C102—C103—C104—C105	179.97 (19)	C217—C216—C222—C221	1.0 (3)
C108—C103—C104—C105	0.4 (3)	C217—C216—C222—C223	−178.1 (2)
C103—C104—C105—C106	0.6 (3)	C220—C221—C222—C216	−0.9 (3)
C104—C105—C106—C107	−1.0 (4)	C220—C221—C222—C223	178.2 (2)
C105—C106—C107—C108	0.4 (4)	C110—C111—C112—C113	−0.5 (4)
C102—C103—C108—C109	−0.2 (3)	C114—C113—C112—C111	−0.4 (4)
C104—C103—C108—C109	179.44 (18)	C120—C118—N1—C101	13.7 (3)
C102—C103—C108—C107	179.46 (18)	C117—C118—N1—C101	−166.66 (17)
C104—C103—C108—C107	−0.9 (3)	C102—C101—N1—C118	59.0 (2)
C106—C107—C108—C109	−179.8 (2)	P1—C101—N1—C118	−171.63 (14)
C106—C107—C108—C103	0.6 (3)	C220—C218—N1B—C201	170.83 (16)
C103—C108—C109—C110	0.1 (3)	C217—C218—N1B—C201	−8.4 (3)
C107—C108—C109—C110	−179.5 (2)	C202—C201—N1B—C218	−62.0 (2)
C108—C109—C110—C111	179.10 (19)	P1B—C201—N1B—C218	168.22 (14)
C108—C109—C110—C115	0.3 (3)	P1—O2—C124—C125	76.8 (12)
C109—C110—C111—C112	−177.8 (2)	P1—O2B—C324—C325	−97 (3)
C115—C110—C111—C112	1.0 (3)	C227—C226—O23—P1B	95.3 (3)
C112—C113—C114—C115	0.8 (3)	C273—C126—C127—O3	−162 (6)
C103—C102—C115—C114	−178.18 (17)	P1—O3—C127—C126	−172.0 (5)
C101—C102—C115—C114	1.2 (3)	P1—O3B—C273—C126	165 (3)
C103—C102—C115—C110	0.7 (2)	C127—C126—C273—O3B	−1(6)
C101—C102—C115—C110	−179.88 (15)	P1B—O22—C224—C225	168.1 (8)
C113—C114—C115—C102	178.57 (19)	P1B—O22B—C229—C425	−179.2 (8)
C113—C114—C115—C110	−0.3 (3)	C127—O3—P1—O1	−10.1 (10)
C109—C110—C115—C102	−0.7 (3)	C127—O3—P1—O2B	−136.1 (12)
C111—C110—C115—C102	−179.51 (17)	C127—O3—P1—O2	−135.4 (9)
C109—C110—C115—C114	178.23 (18)	C127—O3—P1—O3B	58 (4)
C111—C110—C115—C114	−0.5 (3)	C127—O3—P1—C101	119.5 (9)
C122—C116—C117—C118	−0.6 (3)	C324—O2B—P1—O1	−8(3)
C116—C117—C118—N1	−179.37 (18)	C324—O2B—P1—O3	112 (2)
C116—C117—C118—C120	0.3 (3)	C324—O2B—P1—O2	−66 (3)
N1—C118—C120—C121	179.37 (17)	C324—O2B—P1—O3B	115 (2)
C117—C118—C120—C121	−0.3 (3)	C324—O2B—P1—C101	−140.3 (19)
C118—C120—C121—C122	0.5 (3)	C124—O2—P1—O1	−88.8 (7)
C120—C121—C122—C116	−0.8 (3)	C124—O2—P1—O3	38.7 (8)
C120—C121—C122—C123	179.8 (2)	C124—O2—P1—O2B	41 (3)
C117—C116—C122—C121	0.8 (3)	C124—O2—P1—O3B	42.6 (13)
C117—C116—C122—C123	−179.8 (2)	C124—O2—P1—C101	150.8 (7)
N1B—C201—C202—C203	139.97 (16)	C273—O3B—P1—O1	26 (4)
P1B—C201—C202—C203	−93.51 (16)	C273—O3B—P1—O3	−92 (5)
N1B—C201—C202—C215	−40.3 (2)	C273—O3B—P1—O2B	−106 (4)
P1B—C201—C202—C215	86.19 (17)	C273—O3B—P1—O2	−106 (3)
C215—C202—C203—C204	−177.95 (16)	C273—O3B—P1—C101	147 (3)
C201—C202—C203—C204	1.8 (2)	N1—C101—P1—O1	48.50 (15)
C215—C202—C203—C208	2.6 (2)	C102—C101—P1—O1	179.49 (12)
C201—C202—C203—C208	−177.73 (14)	N1—C101—P1—O3	−82.5 (2)
C202—C203—C204—C205	−178.87 (18)	C102—C101—P1—O3	48.5 (3)
C208—C203—C204—C205	0.6 (3)	N1—C101—P1—O2B	−173.6 (10)
C203—C204—C205—C206	−0.8 (3)	C102—C101—P1—O2B	−42.6 (10)
C204—C205—C206—C207	0.2 (4)	N1—C101—P1—O2	168.2 (2)
C205—C206—C207—C208	0.6 (3)	C102—C101—P1—O2	−60.9 (3)
C206—C207—C208—C209	179.4 (2)	N1—C101—P1—O3B	−69.1 (19)
C206—C207—C208—C203	−0.7 (3)	C102—C101—P1—O3B	61.9 (19)
C202—C203—C208—C209	−0.5 (2)	C224—O22—P1B—O21	7.4 (14)
C204—C203—C208—C209	179.98 (17)	C224—O22—P1B—O23	130.3 (12)
C202—C203—C208—C207	179.67 (16)	C224—O22—P1B—O22B	−53.0 (19)
C204—C203—C208—C207	0.1 (2)	C224—O22—P1B—C201	−123.9 (12)
C207—C208—C209—C210	178.13 (17)	C226—O23—P1B—O21	12.5 (2)
C203—C208—C209—C210	−1.7 (3)	C226—O23—P1B—O22	−115.1 (4)
C208—C209—C210—C211	−178.03 (17)	C226—O23—P1B—O22B	−116.3 (4)
C208—C209—C210—C215	1.7 (3)	C226—O23—P1B—C201	137.7 (2)
C209—C210—C211—C212	179.3 (2)	C229—O22B—P1B—O21	−65.1 (16)
C215—C210—C211—C212	−0.5 (3)	C229—O22B—P1B—O22	62 (2)
C210—C211—C212—C213	−0.2 (3)	C229—O22B—P1B—O23	65.7 (16)
C211—C212—C213—C214	0.7 (4)	C229—O22B—P1B—C201	174.9 (14)
C212—C213—C214—C215	−0.5 (3)	N1B—C201—P1B—O21	−58.41 (14)
C203—C202—C215—C214	176.93 (16)	C202—C201—P1B—O21	170.46 (12)
C201—C202—C215—C214	−2.8 (2)	N1B—C201—P1B—O22	76.4 (3)
C203—C202—C215—C210	−2.5 (2)	C202—C201—P1B—O22	−54.7 (4)
C201—C202—C215—C210	177.80 (14)	N1B—C201—P1B—O23	176.40 (12)
C213—C214—C215—C202	−179.64 (18)	C202—C201—P1B—O23	45.27 (15)
C213—C214—C215—C210	−0.2 (3)	N1B—C201—P1B—O22B	58.4 (5)
C209—C210—C215—C202	0.4 (2)	C202—C201—P1B—O22B	−72.7 (5)
C211—C210—C215—C202	−179.83 (16)		

### Hydrogen-bond geometry (Å, °)

**Table:**

*D*—H···*A*	*D*—H	H···*A*	*D*···*A*	*D*—H···*A*
N1—H1···O1^i^	0.98	2.02	2.990 (2)	170
N1B—H1B···O21^ii^	0.90	2.15	3.016 (2)	163

Symmetry codes: (i) −*x*+1, −*y*+1, −*z*+1; (ii) −*x*+1, −*y*, −*z*.
